# Caspase-11-dependent pyroptosis of lung epithelial cells protects from melioidosis while caspase-1 mediates macrophage pyroptosis and production of IL-18

**DOI:** 10.1371/journal.ppat.1007105

**Published:** 2018-05-23

**Authors:** Jinyong Wang, Manoranjan Sahoo, Louis Lantier, Jonathan Warawa, Hector Cordero, Kelly Deobald, Fabio Re

**Affiliations:** 1 Department of Microbiology and Immunology, Rosalind Franklin University of Medicine and Science, North Chicago, Illinois, United States of America; 2 Department of Microbiology and Immunology, University of Louisville, Louisville, Kentucky, United States of America; Stanford University School of Medicine, UNITED STATES

## Abstract

Infection with *Burkholderia pseudomallei* or *B*. *thailandensis* triggers activation of the NLRP3 and NLRC4 inflammasomes leading to release of IL-1β and IL-18 and death of infected macrophages by pyroptosis, respectively. The non-canonical inflammasome composed of caspase-11 is also activated by these bacteria and provides protection through induction of pyroptosis. The recent generation of *bona fide* caspase-1-deficient mice allowed us to reexamine in a mouse model of pneumonic melioidosis the role of caspase-1 independently of caspase-11 (that was also absent in previously generated *Casp1*^*-/-*^ mice). Mice lacking either caspase-1 or caspase-11 were significantly more susceptible than wild type mice to intranasal infection with *B*. *thailandensis*. Absence of caspase-1 completely abolished production of IL-1β and IL-18 as well as pyroptosis of infected macrophages. In contrast, in mice lacking caspase-11 IL-1β and IL-18 were produced at normal level and macrophages pyroptosis was only marginally affected. Adoptive transfer of bone marrow indicated that caspase-11 exerted its protective action both in myeloid cells and in radio-resistant cell types. *B*. *thailandensis* was shown to readily infect mouse lung epithelial cells triggering pyroptosis in a caspase-11-dependent way *in vitro* and *in vivo*. Importantly, we show that lung epithelial cells do not express inflammasomes components or caspase-1 suggesting that this cell type relies exclusively on caspase-11 for undergoing cell death in response to bacterial infection. Finally, we show that IL-18’s protective action in melioidosis was completely dependent on its ability to induce IFNγ production. In turn, protection conferred by IFNγ against melioidosis was dependent on generation of ROS through the NADPH oxidase but independent of induction of caspase-11. Altogether, our results identify two non-redundant protective roles for caspase-1 and caspase-11 in melioidosis: Caspase-1 primarily controls pyroptosis of infected macrophages and production of IL-18. In contrast, caspase-11 mediates pyroptosis of infected lung epithelial cells.

## Author summary

*Burkholderia pseudomallei* is a bacterium that infect macrophages and other cell types and causes a diseases called melioidosis. Inflammasomes are multiprotein complexes that control activation of the proteases caspase-1 and caspase-11 resulting in production of the inflammatory mediators IL-1β and IL-18 and death of infected cells. Mice deficient of caspase-1 or caspase-11 are more susceptible to infection with *B*. *pseudomallei* or the closely related *B*. *thailandensis*. Here we show that absence of caspase-1 completely abolished production of IL-1β and IL-18 as well as death of macrophages infected with *B*. *thailandensis*. In contrast, in the highly susceptible caspase-11-deficient mice, IL-1β and IL-18 production and macrophages death were not significantly affected. Rather, absence of caspase-11 abolished death of infected lung epithelial cells. Taken together, our results show that caspase-1 and caspase-11 have non-redundant protective roles in melioidosis: Caspase-1 primarily controls cell death of infected macrophages and production of IL-18. In contrast, caspase-11 mediates cell death of infected lung epithelial cells.

## Introduction

*Burkholderia pseudomallei* is a Gram-negative flagellated bacterium that causes melioidosis, a diseases endemic to South-East Asia and other tropical regions and the most common cause of pneumonia-derived sepsis in Thailand [1, 2]. Due to global warming and increased international travel, cases of melioidosis are increasingly being reported outside the endemic areas. *B*. *pseudomallei* infection can be contracted through ingestion, inhalation, or subcutaneous inoculation and leads to broad-spectrum disease forms including pneumonia, septicemia, and organ abscesses. Although not pathogenic to humans, *Burkholderia thailandensis* possesses several of the *B*. *pseudomallei* virulence factors, causes morbidity and mortality in mice, and is often used as a model for melioidosis [3–5]. Following infection of macrophages and other non-phagocytic cell types, *Burkholderia* is able to escape the phagosome and invade and replicate in the host cell cytoplasm. Macrophages and IFNγ have been shown to play a critical role in protection from melioidosis [6–8]and several *B*. *pseudomallei* virulence factors have been identified. Analysis of mouse strains with different susceptibility to *B*. *pseudomallei* infection indicates that the early phases of the infection are crucial for survival, emphasizing the necessity for better understanding of innate immune responses during melioidosis.

*Burkholderia* has been shown to activate TLR2, TLR4, and TLR5 in epithelial reporter cell line [9]. Interestingly, while *Myd88*^*-/-*^ mice are highly susceptible to *B*. *pseudomallei* infection [10], *Tlr4*^*-/-*^ mice have similar resistance to wild type (WT) mice but *Tlr2*^*-/-*^ mice showed reduced mortality [11] indicating that MyD88-dependent pathways may play opposite role in melioidosis. This notion is supported by our previous works that showed that IL-18 was protective in melioidosis while IL-1β was deleterious because of excessive neutrophils recruitment to the lung and tissue damage due to release of neutrophil elastase [12, 13].

Caspase-1 has been shown to be protective against *Burkholderia* infections [14]. Production of IL-1β and IL-18 in melioidosis is regulated by activation of caspase-1 downstream of the NLRP3 inflammasome while activation of the NLRC4 inflammasome triggers the pyroptotic cell death process [12, 15]. A potentially confounding factor that affects all the works that examined the role of caspase-1 in melioidosis is that those studies relied on caspase-1-deficient mice that also lacked caspase-11. The non-canonical inflammasome composed of caspase-11 (encoded by *Casp4*) has also been shown to play a protective role in melioidosis [16] by recognizing cytoplasmically-located LPS [17, 18]. This process is dependent on priming of macrophages with interferons or TLR ligands. The mechanism through which caspase-1 and caspase-11 initiate pyroptosis is by cleaving gasdermin D, a cellular protein that open pores in the cell membrane [19, 20].

As for infections by most intracellular bacteria, IFNγ is an essential component of the innate immune response to *B*. *pseudomallei* and its absence results in severely decrease resistance to the infection [6–8]. The antimicrobial properties of IFNγ are mediated by several effector mechanisms operating in a variety of cell types. Among the thousand IFN-stimulated genes, IFN-induced GTPases, iNOS, and NADPH oxidase are the most studied and effective antimicrobial effector mechanisms of macrophages. Recently it has been proposed that in the early phase of *Burkholderia* infection caspase-11 may act as an IFNγ-inducible effector mechanism because of its reliance on the IL-18-IFNγ axis for priming, which would place non canonical inflammasome downstream of canonical caspase-1 activation [15].

The function of IFN-inducible effector mechanisms such as iNOS, ROS, Guanylate binding proteins, and caspase-11 in melioidosis has been examined previously [15, 16, 21, 22]. While iNOS and several GBPs do not seem to be required to survive a lethal infection with *B*. *pseudomallei* or *B*. *thailandensis*, absence of NADPH oxidase or caspase-11 renders mice significantly more susceptible. For this reason we decided to determine the relative contribution of caspase-11 and NADPH oxidase to the protection conferred by IFNγ against *B*. *thailandensis* infection. We also revisited the role of caspase-1 independently of caspase-11 using recently generated *bona fide* caspase-1-deficient mice [23]. The results presented here support the following conclusions: first, the main protective role of caspase-1 in melioidosis is to trigger pyroptosis in macrophages and production of IL-18. Second, caspase-11’s function during *B*. *thailandensis* infection is to mediate pyroptosis in lung epithelial cells, rather than in macrophages. Finally, the protective action of IFNγ is mediated by ROS independently of caspase-11.

## Results

### Caspase-1 and caspase-11 are protective in melioidosis

**Fig 1:**
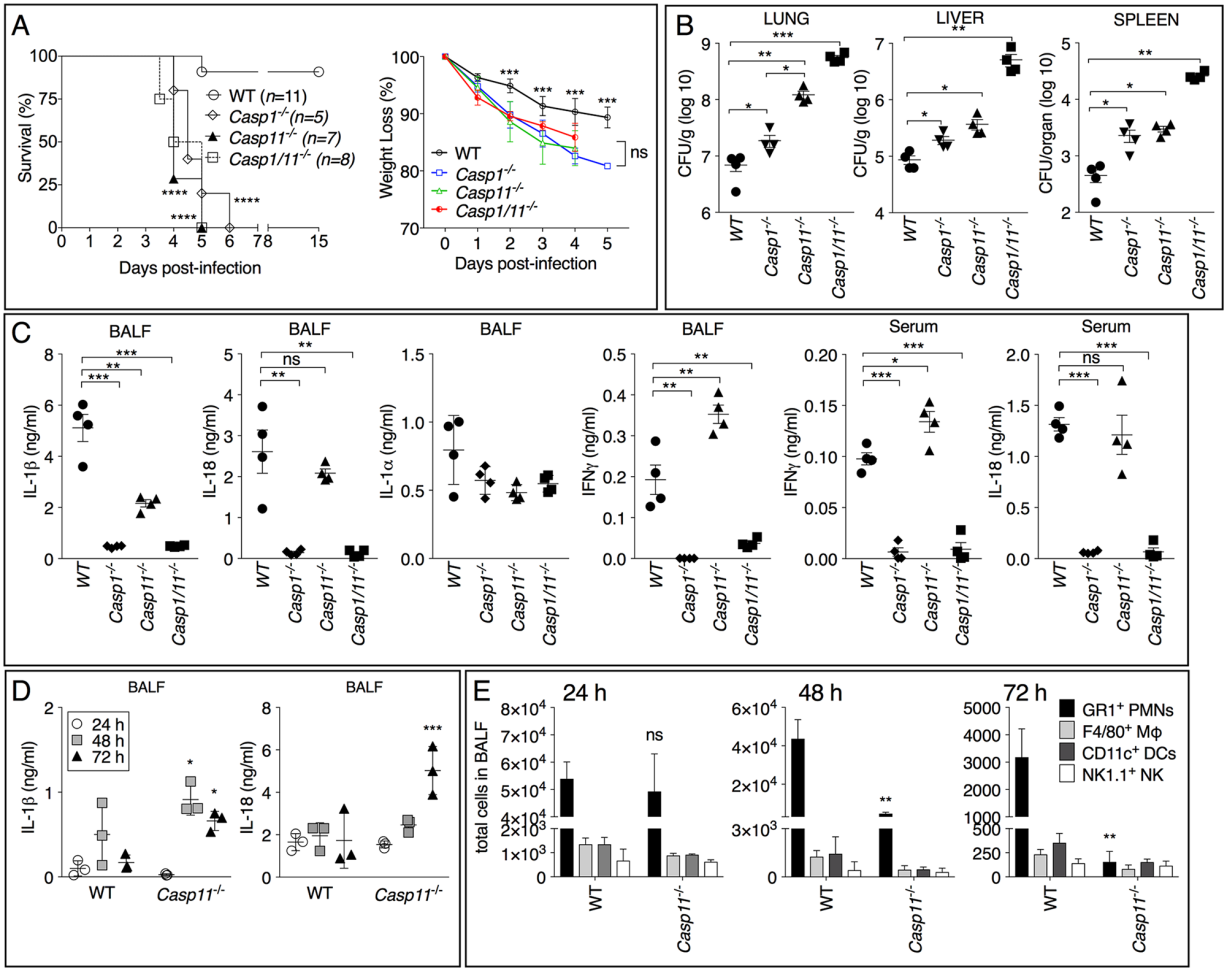
Caspase-1 and caspase-11 are protective in melioidosis. (A) Mice were infected intranasaly with *B*. *thailandensis* (10^5^ CFU) and their survival and body weight were monitored. (B, C) Mice infected with *B*. *thailandensis* (5x10^5^ CFU) were sacrificed 48 hours p.i. and the bacterial burden in organs and cytokines in BALF and serum were measured. (D, E) Mice infected intranasaly with *B*. *thailandensis* (10^5^ CFU) were sacrificed at the shown time points and cytokine levels and cellular infiltrates in BALF were measured. One representative experiment of three (A-C) or two (D, E) is shown. Data are expressed as mean ± S.D. *p<0.05, **p<0.01, ***p<0.001. (A) log rank Kaplan-Meier test, (B) Mann-Whitney *U* test, (C) Unpaired *t-*test, (D, E) One-way ANOVA.

We and others have previously shown that caspase-1 plays a critical role during intranasal or intraperitoneal infection with *Burkholderia* [12–14]. However, those results were obtained using caspase-1-deficient mice that also lacked caspase-11 due to a passenger mutation in the 129 mouse strain. “Pure” caspase-1-deficient mice have been recently generated [23] and for this reason we decided to reexamine the role of caspase-1 and caspase-11 in melioidosis. Analysis of the survival of mice infected intranasally with *B*. *thailandensis* showed that both *Casp1*^*-/-*^ and *Casp11*^*-/-*^ mice were significantly less resistant than WT mice and became moribund within 5 days of infection and had to be euthanized (Fig 1A). The bacterial burdens in different organs were measured at different time points post-infection (p.i.) and in mice infected using different doses and revealed the relative susceptibility of *Casp1*^*-/-*^, *Casp11*^*-/-*^, and *Casp1*^*-/-*^/*Casp11*^*-/-*^ mice (Fig 1B). Mice lacking both caspases had the highest amount of bacteria while mice deficient in caspase-11 were clearly more susceptible than *Casp1*^*-/-*^ mice. This pattern has been previously observed in a study that compared *Casp11*^*-/-*^ mice to mice that lacked both ASC and NLRC4 (used as surrogate for pure caspase-1 deficient mice) [15]. In agreement with the function of caspase-1 in the generation of the mature form of IL-18, this cytokine was absent in BALF or serum of *Casp1*^*-/-*^ mice while was detected at the same level in *Casp11*^*-/-*^ or WT mice (Fig 1C). The levels of IL-1β, a deleterious factor in melioidosis [12, 13], were decreased in *Casp11*^*-/-*^ mice compared to WT mice. However, when *Casp11*^*-/-*^ mice were infected with a lower inoculum and examined at different time points p.i., IL-1β (like IL-18) was detected in their BALF at the same level, or even higher, than in WT mice (Fig 1D). Interestingly, neutrophils were present in the BALF of *Casp11*^*-/-*^ mice in significantly reduced number than in WT mice 48 and 72 hours p.i. (Fig 1E). This phenotype, however, was not observed 24 hours p.i., was not due to impaired production of TNFα, IL-6, KC, or MCP-1, and correlated with the increased bacterial burdens in organs (S1 Fig). Whether absence of caspase-11 negatively affects recruitment, survival, or permanence of neutrophils in the infected lung cannot be determine at present and will be the focus of future studies. Impaired neutrophil recruitment has been previously observed in *Casp11*^*-/-*^ mice during lung infection with *K*.*pneumoniae* [24].

**Fig 2:**
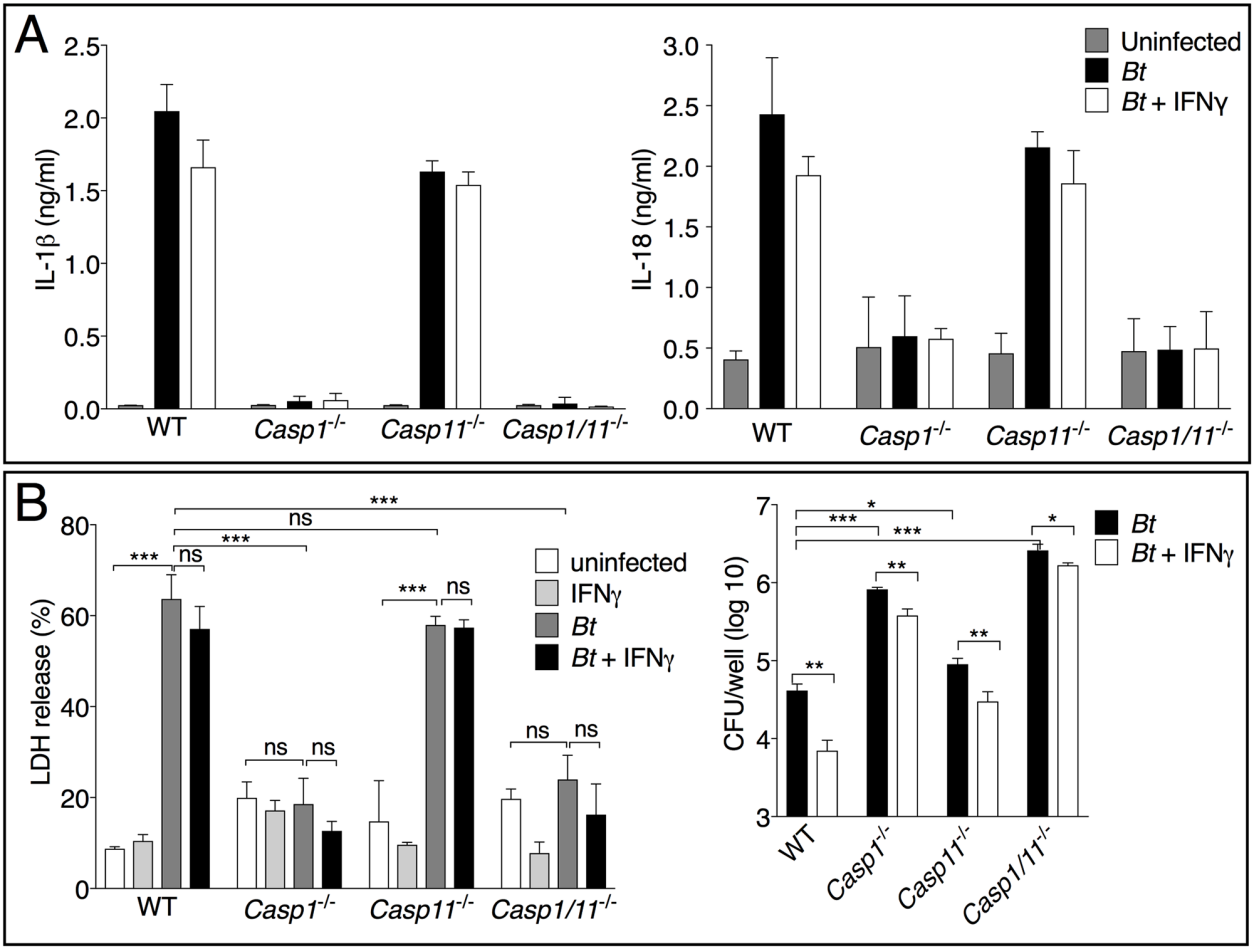
Caspase-1, not caspase-11, controls IL-1β/IL-18 secretion and pyroptosis in BMM infected with *B*. *thailandensis*. BMM were primed O/N with IFNγ (100 ng/ml) and infected with *B*. *thailandensis* (MOI 50). Cytokine secretion (A) and LDH release (B) were measured 4 hours p.i.. Intracellular bacteria replication was measured 7 hours post infection. One representative experiment of four is shown. Data are expressed as mean ± S.D. *p<0.05, **p<0.01, ***p<0.001. (B) One-way ANOVA.

Analysis of bone marrow-derived macrophage (BMM) cultures infected with *B*. *thailandensis* confirmed that IL-1β and IL-18 production is mediated by caspase-1 and not caspase-11 (Fig 2A). Induction of pyroptosis in these cells was primarily dependent on caspase-1 with negligible contribution of caspase-11 (Fig 2B). For these experiments BMM were primed O/N with IFNγ. However, when bone marrow-derived dendritic cells (S2 Fig) or BMM were primed with IFNγ *concomitantly* to infection (see below), caspase-11 contribution to pyroptosis was modest but statistically significant. Intracellular *B*. *thailandensis* replication in BMM inversely correlated to occurrence of pyroptosis with maximal bacterial count in *Casp1*^*-/-*^ and *Casp1/Casp11*^*-/-*^ cells (Fig 2B). *B*. *thailandensis* replication in *Casp11*^*-/-*^ cells was moderately higher than in WT cells. Interestingly, IFNγ priming of cells significantly decreased bacteria replication in all strains emphasizing the importance of inflammasomes-independent microbicidal mechanisms activated by IFNγ (see below). Although *B*. *thailandensis* has been shown to serve as a useful model for melioidosis, it is not pathogenic in humans and differs in many aspects from the virulent *B*. *pseudomallei*. Therefore, it was important to determine the role of caspase-11 even during infection with *B*. *pseudomallei*. As shown in S3 Fig, two light emitting *B*. *pseudomallei* clinical isolates robustly replicated in *Casp1*^*-/-*^*/Casp11*^*-/-*^ macrophages but to a much lower degree in WT and *Casp11*^*-/-*^ cells, again indicating the prominent role of caspase-1, rather than caspase-11, in restriction of intracellular replication of *Burkholderia* in macrophages.

Taken together, these results conclusively indicate that the increased susceptibility of *Casp1*^*-/-*^ mice to melioidosis is likely due to their inability to produce IL-18 and trigger pyroptosis in myeloid cells. In contrast, the reason for the susceptibility of *Casp11*^*-/-*^ mice remains unclear but does not appear to be due to lack of IL-18 or gross inability to trigger pyroptosis in macrophages.

### Caspase-11 protective role in non-hematopoietic cells during melioidosis

**Fig 3:**
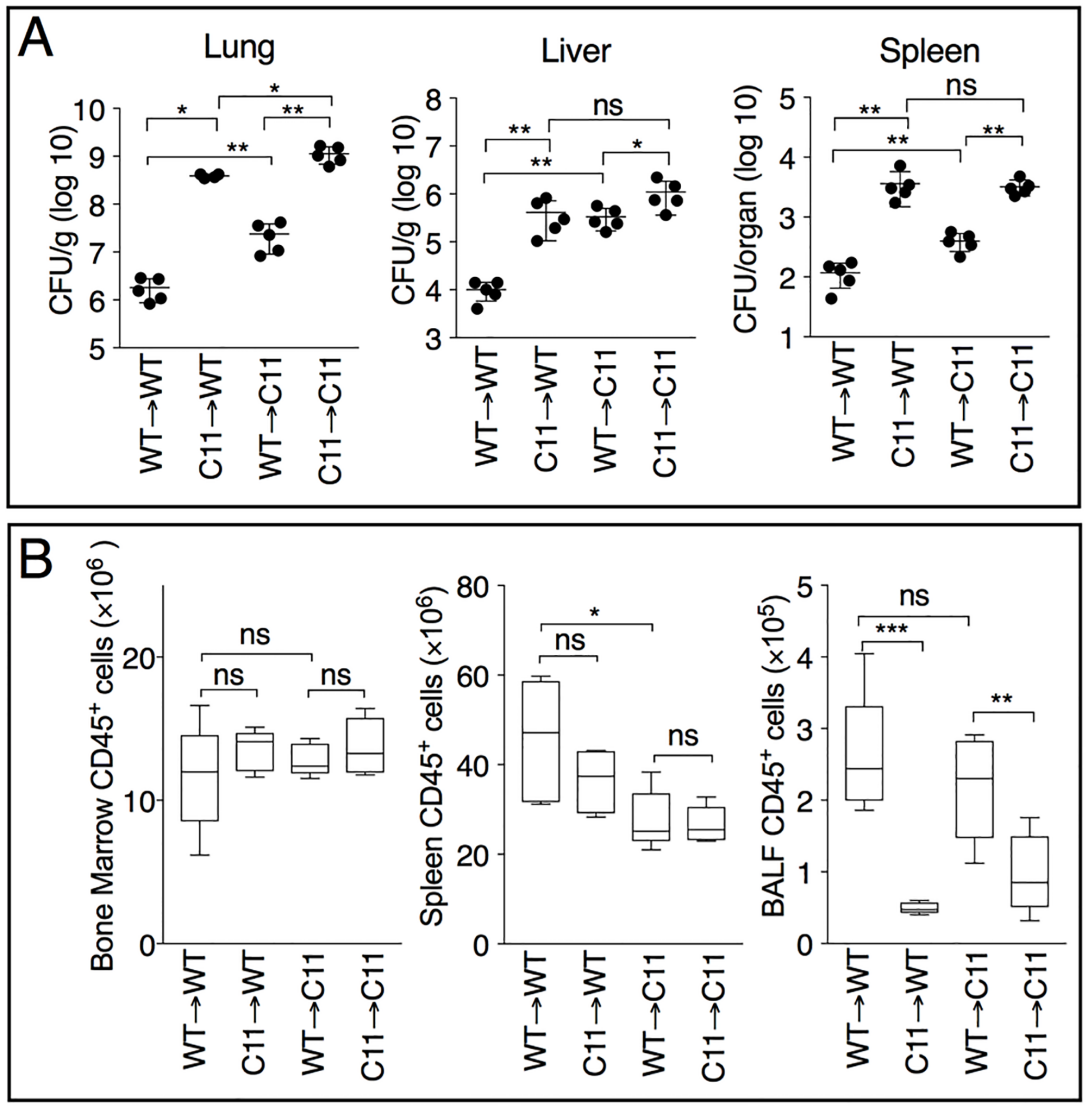
Caspase-11 protective role in hematopoietic and radio-resistant cell types. (A) Mice that underwent adoptive bone marrow transplant were infected with *B*. *thailandensis* (5x10^5^ CFU) and sacrificed 48 hours p.i. to measure the organ bacterial burdens. (B) The total numbers of CD45-positive cells in bone marrow, spleen, and BALF of mice from A were calculated. One representative experiment of two is shown. Data are expressed as mean ± S.D. *p<0.05, **p<0.01, ***p<0.001 (A) Mann-Whitney *U* test, (B) One-way ANOVA.

The fact that pyroptosis of *B*. *thailandensis*-infected macrophages and cytokine processing in these cells appeared mostly dependent on caspase-1 with minor involvement of caspase-11 raised the question of why *Casp11*^*-/-*^ mice appeared so susceptible to melioidosis. Caspase-11 function has been extensively studied in myeloid cells but its role in non-hematopoietic cells has been mostly neglected. To increase our understanding of the role played by Caspase-11 during melioidosis we performed bone marrow transplant experiments. As shown in Fig 3A, the bacterial burden in different organs of WT mice reconstituted with *Casp11*^*-/-*^ bone marrow cells was significantly higher compared to WT mice receiving WT cells, as expected for a hematopoietic role for caspase-11. However, *Casp11*^*-/-*^ mice reconstituted with WT bone marrow cells still had organ bacteria burdens much higher than WT mice indicating that caspase-11 also plays a protective role in the radio-resistant cell compartment. Analysis of bone marrow and spleen cells indicated complete and equally effective reconstitution by both genotypes. (S4 Fig). However, confirming what observed in *Casp11*^*-/-*^ mice (Fig 1E), severely decreased neutrophil numbers were detected into the lung of mice reconstituted with caspase-11-deficient bone marrow (Fig 3B and S4 Fig). These results suggest that caspase-11 plays a protective role not only in hematopoietic cells but also in radio-resistant cell types.

### Caspase-11 controls pyroptosis of lung epithelial cells

**Fig 4:**
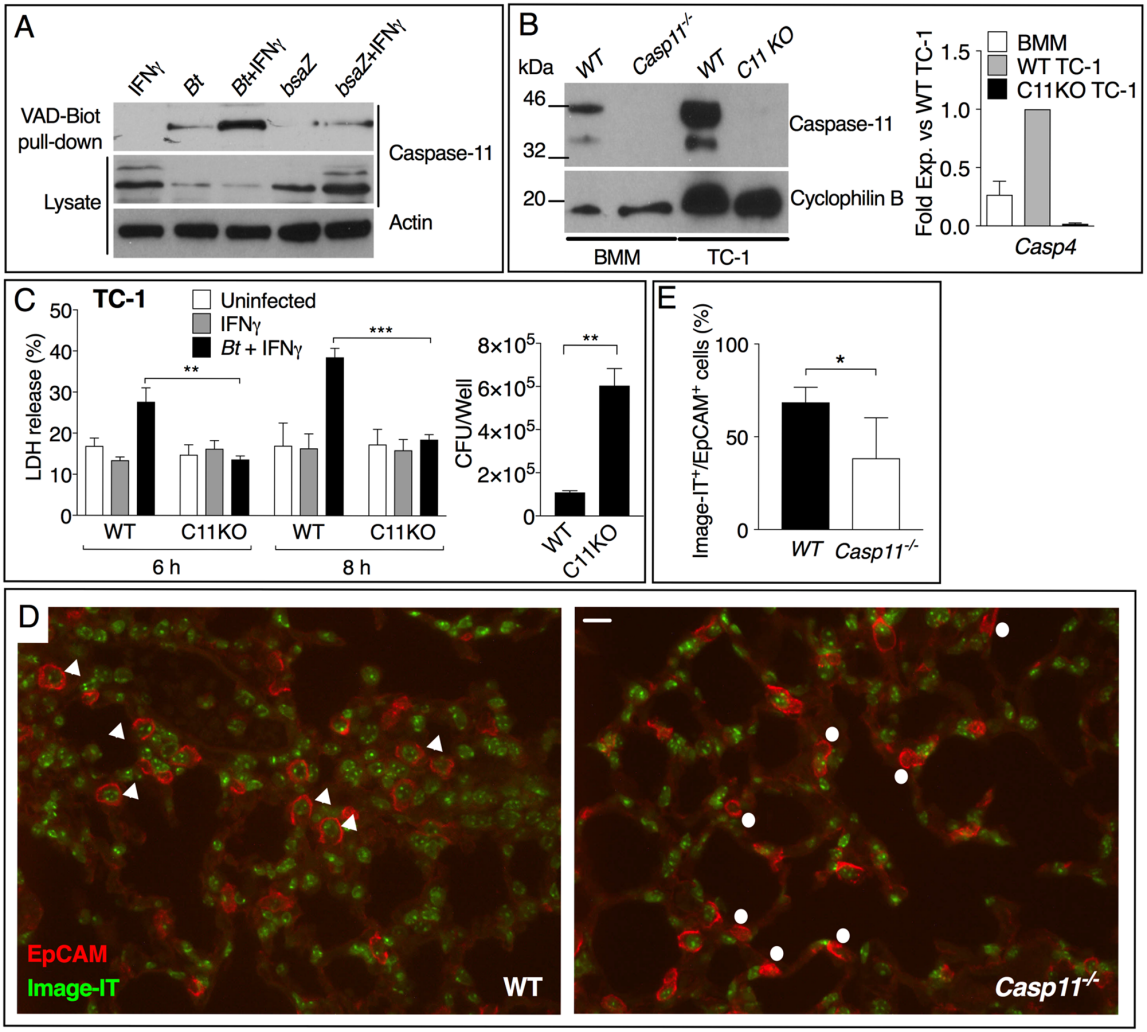
Caspase-11 controls pyroptosis of lung epithelial cells. (A) TC-1 cells were infected with *B*. *thailandensis* or the *bsaZ* mutant in presence or absence of IFNγ. Active caspase-11 was pulled-down from cell lysates using Biotin-VAD-FMK and detected by immunoblot. (B) Caspase-11 expression in control (WT) and knocked-out (C11KO) TC-1 cells. (C) WT and C11KO TC-1 cells were stimulated with IFNγ (100 ng/ml) and infected with *B*. *thailandensis* (MOI 100). LDH release and intracellular bacteria replication were measured 8 hours p.i.. (D, E) Mice infected for 48 hours with *B*. *thailandensis* (5x10^5^ CFU) were treated intranasaly with Image-iT DEAD to stain necrotic cells and euthanized. Lung histological sections were stained with anti-EpCAM antibody to visualize epithelial cells. White arrow heads indicate necrotic epithelial cells, white dots indicate live epithelial cells. One representative experiment of four (A-C) or two (D, E) is shown. *p<0.05, **p<0.01, ***p<0.001 Unpaired *t*-test.

*B*. *thailandensis* can infect several cell types including lung epithelial cells [25, 26]. The mouse lung epithelial cell line TC-1 is often used to study the lung innate immune response to bacteria [27, 28]. TC-1 cells were readily infected with *B*. *thailandensis* (S5A Fig). To test whether caspase-11 can be activated in lung epithelial cells infected with *B*. *thailandensis*, TC-1 cells were incubated with Biotin-VAD-FMK, a cell permeable caspase pseudosubstrate that irreversibly binds to active caspase [29]. As shown in Fig 4A, caspase-11 could be pulled down from *B*. *thailandensis*-infected TC-1 cell lysates using streptavidin agarose. The *B*. *thailandensis bsaZ* mutant, which is unable to escape the phagosome, activated caspase-11 to a much lower degree. Caspase-11 expression in TC-1 cells was strongly induced by TNFα/IFNγ while expression of the canonical inflammasome components NLRP3, NLRC4, ASC, Caspase-1 was not detectable (S5B Fig) suggesting that caspase-11 may be the only pathway available in TC-1 cells to trigger pyroptosis. This was confirmed by knocking-out caspase-11 gene in TC-1 cells using CRISPR/CAS9 technology (Fig 4B). While the control TC-1 cells underwent pyroptosis upon infection with *B*. *thailandensis*, the caspase-11-deficient TC-1 cells were resistant to *B*. *thailandensis*-induced cell death (Fig 4C). Intracellular *B*. *thailandensis* replication proceeded unrestrained in TC-1 cells lacking caspase-11 but was significantly restricted in control TC-1 cells (Fig 4C). TC-1 cells expressed IL-18 mRNA upon treatment with TNFα/IFNγ though IL-18 secretion could not be detected in conditioned culture supernatants (S5C Fig). To test whether cell death of lung epithelial cells occurs *in vivo*, we exposed WT or *Casp11*^*-/-*^ infected mice to a green fluorescent compound that stains the nucleus of necrotic cells *in situ*. Mice were euthanized shortly thereafter and the lungs were fixed in paraformaldehyde. Histological sections were counterstained with the epithelial marker EpCAM and analyzed by immunofluorescence microscopy to visualize necrotic epithelial cells *in situ*. As shown in Fig 4D and 4E, significantly decreased cell death of lung epithelial cells was observed in lung sections of *Casp11*^*-/-*^ mice compared to WT mice. It was recently shown that pyroptotic cells trap intracellular bacteria and are phagocytosed by neutrophils [30]. Our preliminary results (S5D Fig) suggest that pyroptotic epithelial cells encounter the same fate and are phagocytosed by neutrophils and macrophages. Taken together, these results suggest that one of the most critical functions of caspase-11 in melioidosis is to control pyroptosis of lung epithelial cells.

### IFNγ is necessary and sufficient for the protective effect of IL-18 in melioidosis

**Fig 5:**
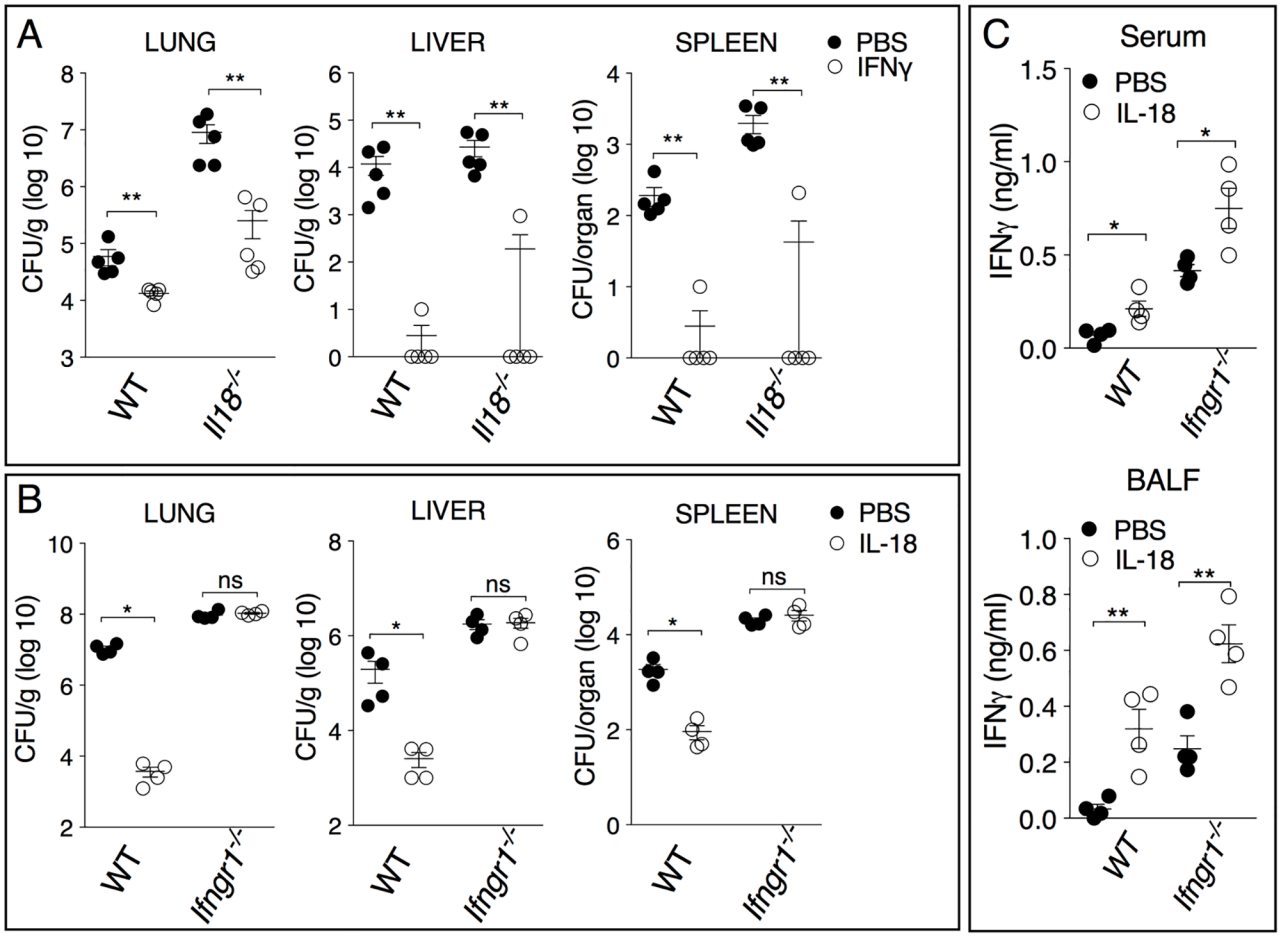
IFNγ is necessary and sufficient for the protective effect of IL-18 in melioidosis. Mice of shown genotype were infected intranasaly with *B*. *thailandensis* (5x10^5^ CFU) and treated with IFNγ (A) or IL-18 (B). Mice were sacrificed 48 hours p.i. and the organ bacterial burdens were measured. (C) IFNγ was measured in the BALF and serum of mice from B. One representative experiment of two is shown. Data are expressed as mean ± S.D. *p<0.05, **p<0.01. (A, B) Mann-Whitney *U* test (C) Unpaired *t*-test.

IL-18 is a potent inducer of IFNγ, a cytokine required to survive infection with *B*. *thailandensis* [12, 31]. In agreement with this activity of IL-18 and with previous works from our and others labs, IFNγ production was severely decreased in *Casp1*^*-/-*^ mice but not *Casp11*^*-/-*^ mice (Fig 1C) suggesting lack of IFNγ as a possible mechanism to explain the protective effect of IL-18. Confirming this hypothesis, administration of recombinant IFNγ significantly reduced organ bacteria burdens in *Il18*^*-/-*^ mice infected with a lethal dose of *B*. *thailandensis* (Fig 5A) showing that IFNγ is *sufficient* to mediate the protective action of IL-18. However, IL-18 performs other functions and, therefore, we asked whether IFNγ was *necessary* for the protective action of IL-18. As shown in Fig 5B, administration of recombinant IL-18 significantly decreased organ bacterial burdens in WT mice but not in *Ifngr1*^*-/-*^ mice. IL-18 treatment induced IFNγ in both mouse strains (Fig 5C). Taken together, these results demonstrate that IFNγ is necessary and sufficient to mediate the protective action of IL-18.

### IFNγ protection in melioidosis depends on ROS generation

We next turned our attention on the role of IFNγ in melioidosis. A number of studies including from our group [6–8, 12], have demonstrated the protective role of IFNγ during *B*. *thailandensis* infection though the mechanism of protection remains undefined. IFNγ is known to activate several microbicidal mechanisms that are critical for killing intracellular bacteria, including different families of GTPases, NRAMP1, NADPH oxidases and iNOS. Recent work suggested that caspase-11 should also be considered as an IFNγ-inducible mechanism [15]. Because both ROS and caspase-11 have been shown to be protective against *B*. *thailandensis* infection while iNOS, NRAMP1, or GBP do not appear to play a significant role in the innate immune response against this bacterium [15, 21, 22, 32], we decided to determine to what degree the protective action of IFNγ in melioidosis is mediated by either ROS or caspase-11.

**Fig 6:**
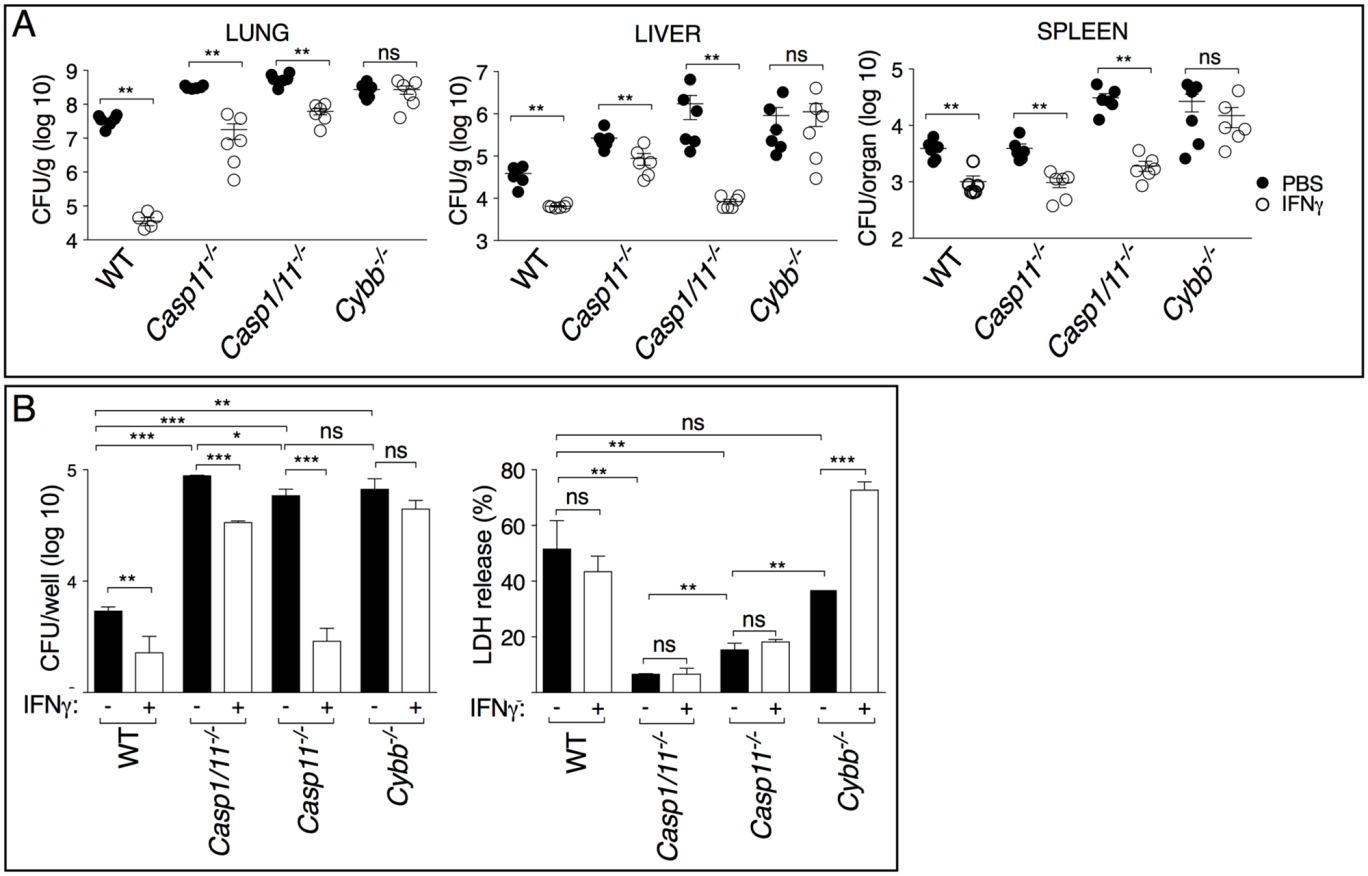
IFNγ protection in melioidosis depends on ROS generation. (A) Mice were infected intranasaly with *B*. *thailandensis* (10^5^ CFU) and treated with IFNγ. Mice were sacrificed 48 hours p.i. and the organ bacterial burdens were measured. (B) BMM were infected with *B*. *thailandensis* (MOI 50) in presence or absence of IFNγ (100 ng/ml)). Cells were lysed 4 hours later and intracellular bacteria growth was measured (left panel). Induction of pyroptosis was measured as LDH release in conditioned supernatants (right panel). One experiment representative of three is shown. Data are expressed as mean ± S.D. *p<0.05, **p<0.01, ***p<0.001 (A) Mann-Whitney *U* test (B) One-way ANOVA.

As shown in Fig 6A, administration of recombinant IFNγ to intranasally infected mice significantly reduced the organ bacterial burdens in WT, *Casp1*^*-/-*^*/Casp11*^*-/-*^ and *Casp11*^*-/-*^ mice but not in *Cybb*^*-/-*^ mice (deficient in the gp91 subunit of the NADPH oxidase), suggesting that production of ROS is an essential microbicidal mechanism triggered by IFNγ against *B*. *thailandensis*.

The importance of ROS as mediator of IFNγ protection was also observed in culture of BMMs infected with *B*. *thailandensis* (Fig 6B). Intracellular bacteria replication was drastically higher in *Casp1*^*-/-*^*/Casp11*^*-/-*^ and *Casp11*^*-/-*^ macrophages compared to WT cells and correlated with the decreased pyroptosis in cells lacking either caspase. Intracellular *B*. *thailandensis* replication was also elevated in *Cybb*^*-/-*^ macrophages but this was not due to decreased pyroptosis, which was not significantly different than in WT cells. Treatment with IFNγ significantly restricted bacteria replication in WT, *Casp1*^*-/-*^*/Casp11*^*-/-*^, and *Casp11*^*-/-*^ cells but, importantly, not in *Cybb*^*-/-*^ cells. The decreased bacteria replication was not due to higher rate of pyroptosis, which was not significantly affected by treatment with IFNγ. Taken together, these results suggest that production of ROS plays a predominant role in the antimicrobial action of IFNγ.

**Fig 7:**
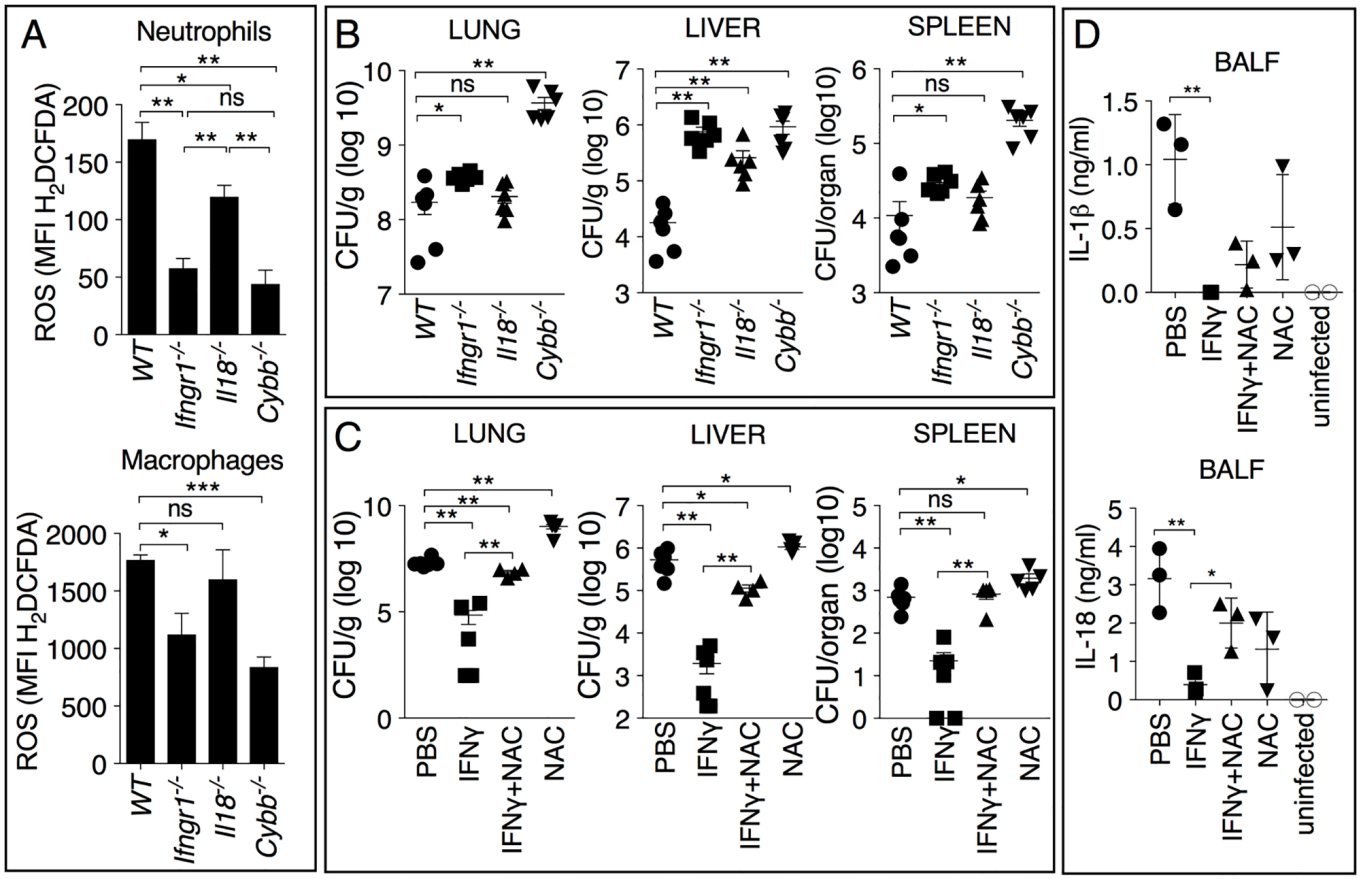
IFNγ stimulates ROS production that protects from *B*. *thailandensis* infection. Mice were infected intranasaly with *B*. *thailandensis* (10^5^ CFU). (A) ROS generation was measured *ex vivo* in neutrophils or macrophages obtained from BALF of mice of shown genotype 14 or 48 hours p.i., respectively. (B) Mice were sacrificed 48 hours p.i. and the organ bacterial burdens were measured. (C, D) Wild type mice were infected intranasaly with *B*. *thailandensis* (10^5^ CFU) and treated daily with IFNγ or NAC. Mice were sacrificed 48 hours p.i. and the organ bacterial burdens (C) or cytokine levels in BALF (D) were measured. One representative experiment of two is shown. Data are expressed as mean ± S.D. *p<0.05, **p<0.01, ***p<0.001 (A, D) Unpaired *t*-test, (B, C) Mann-Whitney *U* test.

Confirming the protective role of NADPH oxidase downstream of IFNγ, *ex vivo* generation of ROS was significantly impaired in neutrophils or macrophages obtained from the BALF of infected *Il18*^*-/-*^ or *Ifngr1*^*-/-*^ mice 14 hours or 48 hours p.i. (Fig 7A). The organ bacterial burdens in these mice inversely correlated with the amount of ROS produced (Fig 7B). Importantly, administration of the antioxidant N-acetyl-cysteine (NAC) dissipated the protective effect of exogenous IFNγ administration (Fig 7C), again reinforcing the notion that ROS is a major mediator of the protective effect of IFNγ in melioidosis. NAC treatment had no effect on the production of IL-1β or IL-18, whose BALF levels correlated with the bacterial burdens (Fig 7D).

## Discussion

The innate immune response to lung infection with *Burkholderia* species has been examined in a few papers but much remains to be learned. Here we have analyzed the role of the canonical and non-canonical inflammasomes and of the IL-18-IFNγ axis in a mouse model of melioidosis. We and others have previously shown that processing and secretion of the mature form of IL-1β and IL-18 in response to *Burkholderia* infection was dependent on caspase-1 [12, 14, 15]. The caveat of those studies is that they were performed using mice that also lacked caspase-11. The recent generation of *bona fide* caspase-1-deficient mice [23] allowed us to examine for the first time the role of this caspase independently of concomitant absence of caspase-11. Our data conclusively demonstrate that processing and secretion of IL-1β and IL-18 in response to *B*. *thailandensis* infection *in vivo* or *in vitro* is completely dependent on caspase-1 but unaffected by absence of caspase-11. The fact that IL-1β secretion is not reduced in absence of caspase-11 also indicates that activation of the NLRP3 inflammasome, which we previously showed exclusively controls IL-1β and IL-18 secretion in response to *Burkholderia* species infection [12, 13], does not occur as a consequence of caspase-11-mediated pyroptosis and potassium efflux, as in other circumstances [33]. Our results also show that pyroptosis of macrophages infected with *B*. *thailandensis* or *B*. *pseudomallei* and restriction of intracellular bacteria replication is primarily mediated by caspase-1 with minor involvement of caspase-11. Previous works have attributed a more prominent role to caspase-11 in the pyroptosis of *B*. *thailandensis*-infected BMM [15]. It should be noted that those studies relied on *Asc*^*-/-*^*Nlrc4*^*-/-*^ cells or *Casp1*^*-/-*^*/Casp11*^*-/-*^ cells reconstituted with transgenic human caspase-4 as proxy of *bone fide* caspase-1 deficient cells, two models that may not faithfully represent caspase-1 absence. Our results also indicate that experimental variables, such as the length of IFNγ priming, may lead to discordant conclusions regarding the involvement of caspase-11 in the pyroptosis of myeloid cells. In fact, it has been proposed that caspase-11 may function as a back-up mechanism to trigger pyroptosis in situations where caspase-1 may be inactivated [34].

The most important result of our study was obtained through bone marrow adoptive transfer experiments and the analysis of the role of caspase-11 in epithelial cell. Our data show that while cell death in *B*. *thailandensis*-infected macrophages occurred primarily through caspase-1, with caspase-11-dependent pathway playing a secondary role, pyroptosis of lung epithelial cells was exclusively dependent on caspase-11 and efficiently restricted intracellular *B*. *thailandensis* replication in these cells. Interestingly, lung epithelial cells do not express canonical inflammasome components and therefore depend exclusively on caspase-11 for induction of pyroptosis. It is surprising to observe that *Casp1*^*-/-*^ mice that are unable to release mature IL-18/IL-1β or trigger pyroptosis in myeloid cells appear as susceptible (if not more, Fig 1B) as *Casp11*^*-/-*^ mice that are sufficient for both functions. At face value, this result would attribute equal importance to pyroptosis triggered by caspase-11 in epithelial cells and to that triggered by caspase-1 in myeloid cells plus IL-18 production, a notion previously underappreciated. Thus, caspase-11 dependent pyroptosis of infected lung epithelial cells may be the main protective mechanism triggered by the non-canonical inflammasome in melioidosis. The non-canonical inflammasome was recently shown to restrict *S*. *typhimurium* replication in intestinal epithelial cells [35]. Caspase-11 has also been shown to control pyroptosis of endothelial cells during endotoxemia-induced lung injury [36]. Thus, it is conceivable that activation of caspase-11 in cell types other than myeloid or epithelial cells may also play a protective role in melioidosis, an issue we will examine in future studies.

Extending our previous work, we show here that IL-18’s protective action in melioidosis is exclusively dependent on its ability to induce IFNγ. This is an important result because IL-18, in addition to being a strong inducer of IFNγ, also has many other activities. In fact, it has been shown that IL-18 can protect from Streptococcal infections independently of IFNγ [37]. The fact that IFNγ appeared to be indispensable to survive *B*. *thailandensis* infection prompted us to investigate the downstream effector mechanisms triggered by IFNγ and responsible for the protection. We concentrated on caspase-11 and the NADPH oxidase because both pathways were already known to provide protection from *B*. *thailandensis* infection and because both are IFNγ-inducible, though it was unclear which one contributed more prominently to the IFNγ protective effect. Our data show that *in vitro* and *in vivo* the protective action of IFNγ is dependent on production of ROS through the NADPH oxidase system while caspase-11 was dispensable. A previous study has concluded that IFNγ primes caspase-11 in vivo to protect from melioidosis [15]. Although that study ruled out contributions from iNOS and GBP encoded on chromosome 3, the role of NADPH oxidase was not examined. Moreover, that study used a strain of *B*. *thailandensis* that has been passaged into *Casp1*^*-/-*^*/Casp11*^*-/-*^ mice to acquire higher virulence and used the intraperitoneal infection route, rather than the intranasal one, as in our study. Although it is clear that caspase-11 priming is a necessary step for the function of this molecule, it should be pointed out that several inflammatory stimuli, including TLR agonists produced by *B*. *thailandensis*, can prime caspase-11 as efficiently as IFNγ. Interestingly, it was shown that human caspase-4 does not require IFNγ priming in vivo [15].

The results presented here also indicate that caspase-11 may control recruitment of neutrophils to the infected lung. A similar observation has been previously reported during infection with *K*. *pneumoniae* [24]. The reason for the impaired inflammatory response of *Casp11*^*-/-*^ mice is unclear and actively pursued in our lab. Preliminary analysis failed to detect defective production of the main neutrophil-specific chemotactic factors. It is conceivable that chemotactic alarmins released by pyroptotic epithelial cells may be the missing factor in *Casp11*^*-/-*^ infected mice. Whether neutrophils are effective against *Burkholderia* species is an unresolved issue. We and others have shown that neutrophils are not very effective against this bacterium and that excessive neutrophil recruitment to the infected lung becomes deleterious due to tissue damage caused by release of neutrophil elastase [12, 13, 38]. For these reasons, we think it is unlikely that the high susceptibility to melioidosis of *Casp11*^*-/-*^ mice is primarily due to the observed defective neutrophil recruitment.

In conclusion, our results identify non-redundant mechanisms activated by the canonical and the non-canonical inflammasomes that confer host protection in melioidosis: Caspase-1-dependent activation of the IL-18-IFNγ-NADPH oxidase axis and pyroptosis in myeloid cells and caspase-11-dependent pyroptosis of infected lung epithelial cells.

## Materials and methods

### Ethics statement

All the animal experiments described in the present study were conducted in strict accordance with the recommendations in the *Guide for the Care and Use of Laboratory Animals* of the National Institutes of Health. All animal studies were conducted under protocols approved by the Rosalind Franklin University of Medicine and Science Institutional Animal Care and Use Committee (IACUC #B14-17). All efforts were made to minimize suffering and ensure the highest ethical and humane standards.

### Mice

C57BL/6J, B6.SLJ, *Il18*^*-/-*^, *Casp1*^-/-^/*Casp11*^-/-^, *Cybb*^*-/-*^, *and Ifngr1*^*-/-*^ mice were purchased from Jackson lab. *Casp11*^-/-^ mice were provided by Vishva Dixit (Genentech) and *Casp1*^-/-^ mice by Mohamed Lamkanfi (VIB Belgium). All mouse strains were on C57BL/6J genetic background and were bred under specific pathogen-free conditions in the RFUMS animal facility. Age-(8–12 weeks old) and sex-matched animals were used in all experiments. Experimental groups were composed of at least 5 mice, unless stated otherwise.

### Bacteria strains, intranasal infections, and treatments

*B*. *thailandensis* E64 was obtained from ATCC. *B*. *pseudomallei* K96243 and 390b are clinical virulent isolates. Bacteria were grown in Luria broth to mid-logarithmic phase, their titer was determined by plating serial dilutions on LB agar, and stocks were maintained frozen at -80°C in 20% glycerol. For mice infections, frozen stocks were diluted in sterile PBS to the desired titer. Mice were anesthetized using isoflurane and the infectious doses were applied to the nares in 50 μl total volume PBS. Recombinant murine IL-18 (MBL, Nagoya, Japan) was delivered intranasally (1 μg) 6 hours prior to bacterial infection. Two additional IL-18 treatments (1 μg/each) were administered by intraperitoneal injections at 12–15 hours intervals before euthanasia. Recombinant murine IFNγ (Pepro Tech, NJ, USA) was administered by intraperitoneal injections (2 μg) once daily for two days. In other experiments, 1 μg IFNγ was administered by intraperitoneal injections once daily for two days in the presence or absence of 10 mg *N-acety-L-cysteine* (NAC, Sigma) delivered at 12–15 hours intervals for 2 days. All cytokines were diluted to desired concentrations with PBS and PBS alone was applied as control.

### Determination of bacteria growth in organs

Organs aseptically collected were weighted and homogenized in 1 ml PBS. Serial dilutions were plated on LB agar plates containing Streptomycin (100 μg/ml) using the Eddy Jet Spiral Plater (Neutec). Bacterial colonies were counted 24 hours later using the Flash & Grow Automated Bacterial Colony Counter (Neutec).

### BALF collection and cytokines measurements

BALF were collected from euthanized mice by intratracheal injection and aspiration of 1 ml PBS. Cytokines levels in tissue culture conditioned supernatants, BALF, or sera were measured by ELISA using the following kits: MCP-1, IFNγ, TNFα, KC, IL-1α, IL-1β, IL-6 (eBioscience), and IL-18 (MBL Nagoya, Japan).

### Flow cytometry

Cells obtained from BALF were counted and stained with anti-CD11b, anti-CD11c, anti-F4/80, anti-Ly6G, anti-NK1.1 and acquired with a LSRII BD flow cytometer. For reactive oxygen species (ROS) measurement, BALF were collected and immediately spun down at 300 × g for 10 minutes to collect cells. Cells were loaded with 7 μM freshly prepared 2',7'-dichlorodihydrofluorescein diacetate, H2DCFDA (Molecular Probes) in PBS at 37 °C for 30 minutes. Cells were stained with anti-CD11b, anti-CD11c, anti-Ly6G, and anti-F4/80 for 10 minutes, washed twice with PBS, and immediately acquired using a LSRII BD flow cytometer. The ROS level was assessed by MFI of DCF (an oxidized product of H2DCFDA) using FITC-channel. Data was analyzed using FlowJo (TreeStar, OR, USA) software.

### Western blot

Cell lysates were separated by SDS-PAGE, transferred to PVDF membranes, and probed with anti-Caspase-11 antibody (Abcam, ab180673), anti-β-Actin antibody (Cell signaling, 4967), anti-Cyclophilin B antibody (Abcam, ab178397). HRP-conjugated anti-Rabbit IgG antibody (Sigma, A0545) was used as secondary antibody. Immunoblots were developed using ECL method and exposed to X-ray film.

### BMM and TC-1 pyroptosis and intracellular bacteria growth

Release of LDH in tissue culture media, a reflection of pyroptosis, was measured using the Roche Cytotoxicity Detection Kit (Roche Applied Science, 11644793001). BMM or TC-1 cells were plated in 48-well plates. Bacteria were added to the cell culture and the plates were centrifuged at 300x g for 10 minutes to maximize and synchronize infection and incubated for 30 minutes (BMM) or 2 hours (TC-1) at 37°C. Cells were washed with PBS to remove extracellular bacteria and medium containing kanamycin and gentamicin (200 μg/ml each) was added to inhibit extracellular bacteria growth. Media were collected at 4 and 8 hours post infection for LDH measurement. Cells were lysed in PBS-2% saponin-15% BSA and serial dilutions of the lysates were plated on LB agar plates containing streptomycin (100 μg/ml). For real time cell culture infection with *B*. *pseudomallei*, BMM infected with strain 390B or K96193 (MOI 10) in black 96-well plates were incubated in a 37° C, humidified, 5% CO_2_ atmosphere IVIS Spectrum camera system. Images were captured every 10 min for 10 hr using capture settings of 1 min with medium binning. Grid ROI measurements of Total Flux (p/s) per well were extracted for plotting luminescence of viable bacteria as a function of infection time. All work with *B*. *psuedomallei* was performed under biosafety level-3 (BSL3/ABSL3) containment according to policies and standard operating procedures approved via the University of Louisville Committee on Biocontainment and Restricted Entities, The University of Louisville has been approved for select agent work by the Centers for Disease Control and Prevention.

### Bone marrow transplantation

Bone marrow from 8-weeks old B6.SJL (CD45.1) mice or *Casp-11*^*-/-*^ mice (CD45.2) was harvested and 10^6^ bone marrow cells were injected intravenously into lethally irradiated (1040 rad) B6.SJL or *Casp11*^*-/-*^ mice (8-weeks of age). Chimeric mice were infected five weeks later. Peripheral blood, bone marrow cells, and splenocytes were stained with anti-CD45.1 and anti-CD45.2 (Biolegend) and analyzed by flow cytometry to confirm the efficiency of bone marrow reconstitution.

### TC-1 infection and CRISPR/CAS9

TC-1 were kindly provided by Thomas Kawula (Washington State University) and grown in RPMI1640-10% FCS. To target caspase-11 gene, TC-1 cells were transfected using Effectene reagent (Quiagen) with Caspase-11 CRISPR/CAS9 KO plasmid and caspase-11 HDR plasmid (Santa Cruz sc-419462 and sc-419462-HDR). TC-1 cells were also transfected with Control CRISPR/CAS9 plasmid (sc-418922) as control. Cells positive for GFP and RFP expression were sorted using BD FACSAria II cell sorter and single cell clones isolated. Expression of caspase-11 in different clones was measured by RT-PCR and immunoblot. One clone was selected that lacked caspase-11 protein expression and produced an aberrant *Casp4* mRNA that lacked exons 3, 4, and 5 (S6 Fig).

### Active caspase-11 pull-down

TC-1 cells were seeded into 10 cm tissue culture dishes and infected when confluent with *B*. *thailandensis* or the *bsaZ* mutant (MOI 500) in a final volume 5 ml in the presence or absence of 100 ng/ml IFNγ. Three hours later, cell monolayers were extensively washed with D-PBS to remove extracellular bacteria and incubated for another 4 hours in medium containing gentamicin and kanamycin (200μg/mL). Biotin-VAD-FMK (15 μM, Santa Cruz) was added to the culture medium one hour before lysing cells in RIPA buffer. Active caspase-11 was pulled down by incubation overnight at 4 °C with streptavidin agarose (Sigma Aldrich) and analyzed by Western Blot with rabbit anti-caspase-11 antibody (Abcam, ab180673)

### In vivo detection of lung epithelial cell death

Mice infected with *B*. *thailandensis* (5x10^5^ CFU) for 48 hours were administered intranasally with the Image-iT DEAD Green viability stain (Invitrogen, 1 nmole in 50 μl saline) and euthanized 30 minutes later. Lung were perfused and fixed in 4% paraformaldehyde/PBS and embedded in paraffin. Four-micron sections were stained with anti-EpCam antibody (Abcam, ab213500) followed with Alexa Fluor-647 to label Clara cells and Alveolar Type II pneumocytes and visualized with a Nikon Eclipse 80i Microscope equipped with photometrics coolsnap ES2 imaging system. For quantification of dead cells, up to 400 EpCAM-positive cells were counted in six random fields and scored for nuclear positivity to the green fluorescent viability stain.

### Statistical analysis

All data were expressed as mean ± S.D. Survival curves were compared using the log rank Kaplan-Meier test. Mann-Whitney *U* test, One-way ANOVA Tukey Post-test, or unpaired *t*-test were used for analysis of the rest of data as specified in the figure legends. Significance was set at *p*<0.05.

## Supporting information

S1 Text qPCR. TC-1 cells were lysed using TRIzol. 1ug of total RNA was treated with DNase I and cDNA was generated using random hexamers and SuperScript III First-Strand Synthesis System (Invitrogen). Quantitative PCR was performed using PowerUp SYBR Green Master Mix (Applied Biosystems,) using 2uL of cDNA template per reaction. Values are calculated via the 2^-dCt method for relative expression where dCt is calculated by subtracting the actin Ct value from the corresponding gene Ct value. Fold expression values were calculated via the 2^-ddCt method where dCt values were calculated, as above, and then normalized to the WT TC-1 dCt value, which is graphed at a value of “1” when 2^-ddCt is calculated. Normalized Values: *dCt* = *Gene Ct* − *Actin Ct*. *ddCt* = (*Gene Ct* − *Actin Ct*) − *WT TC Ct*. Relative Expression: *Graphed Value* = 2^−*dCt*^. Fold Expression: *Graphed Value* = 2^−*ddCt*^.
(DOCX)

S1 Fig Role of caspase-11 in melioidosis. Wild type or *Casp-11*^*-/-*^ mice infected intranasaly with *B*. *thailandensis* (10^5^ CFU) were sacrificed at the shown time points and cytokine and chemokines levels in BALF (A) or organ bacterial burdens (B) were measured. Data are expressed as mean ± S.D. *p<0.05, **p<0.01. (A) One-way ANOVA, (B) Unpaired *t*-test.
(TIFF)

S2 Fig Role of caspase-1 and caspase-11 in the pyroptosis of bone marrow derived dendritic cells. BMDC were treated with IFNγ (100 ng/ml) and simultaneously infected with *B*. *thailandensis* (MOI 50). LDH release was measured 6 hours p.i. *p<0.05, **p<0.01. One-way ANOVA.
(TIFF)

S3 Fig Caspase-1, not caspase-11, restricts replication of virulent *B*.*pseudomallei* in BMM. BMM of shown genotype were treated with IFNγ (100 ng/ml) and infected with light emitting *B*.*pseudomallei* clinical isolates 390b (A) or K96243 (B) (MOI 10). Bacteria replication (as measured by light emission) was monitored for 600 minutes post infection. One representative experiment of two is shown.
(TIFF)

S4 Fig Bone marrow adoptive transfer. (A) Efficiency of bone marrow reconstitution was measured in BALF, bone marrow (BM), and PBMC by staining CD45.1- and CD45.2-positive cells. (B) Total number of neutrophils, DCs, and macrophages in BALF of infected mice from Fig 3. (C) IL-1β and IL-18 were measured in BALF of infected mice from Fig 3.
(TIFF)

S5 Fig TC-1 lung epithelial cells. (A) TC-1 cells were infected with GFP-expressing *B*. *thailandensis* (MOI 50). (B) Relative expression of *Casp4 and* canonical inflammasome components in TC-1 cells stimulated with TNFα (50 ng/ml) and IFNγ (100 ng/ml) for 8 hours or in BMM. (C) Expression of *Il18* mRNA or measurement of IL-18 in TC-1 conditioned supernatants. (D) Macrophages and neutrophils obtained from control or infected mice were stained for EpCAM and analyzed by flow cytometry.
(TIFF)

S6 Fig Sequence of *Casp4* cDNA of TC-1 C11KO. Sequence alignment of reference and targeted *Casp4* cDNA showing deletion of exons 3, 4, 5 in TC-1 C11 KO.
(TIF)
