# Supplementary material for: Caspase-11-dependent pyroptosis of lung epithelial cells protects from melioidosis while caspase-1 mediates macrophage pyroptosis and production of IL-18
Source: PLoS Pathog. 2018 May 23;14(5):e1007105. doi: 10.1371/journal.ppat.1007105 (PMC5988316; doi:10.1371/journal.ppat.1007105)
Supplement: S1 Text — TC-1 cells were lysed using TRIzol. 1ug of total RNA was treated with DNase I and cDNA was generated using random hexamers and SuperScript III First-Strand Synthesis System (Invitrogen). Quantitative PCR was performed using PowerUp SYBR Green Master Mix (Applied Biosystems,) using 2uL of cDNA template per reaction. Values are calculated via the 2^-dCt method for relative expression where dCt is calculated by subtracting the actin Ct value from the corresponding gene Ct value. Fold expression values were calculated via the 2^-ddCt method where dCt values were calculated, as above, and then normalized to the WT TC-1 dCt value, which is graphed at a value of “1” when 2^-ddCt is calculated. Normalized Values: dCt = Gene Ct − Actin Ct. ddCt = (Gene Ct − Actin Ct) − WT TC Ct. Relative Expression: Graphed Value = 2−dCt. Fold Expression: Graphed Value = 2−ddCt. (DOCX) [file ppat.1007105.s001.docx]

The following primers were used:

*b-actin*: F-GGCTGTATTCCCCTCCATCG;R-CCAGTTGGTAACAATGCCATGT

*Casp4*: F-CAATGCTGAACGCAGTGACAA; R-TCAGGGGAACAAAGCTTGAGA

*Casp1*: F-CCAAGCTTGAAAGACAAGCCC; R-CCCTGACAGGATGTCTCCAA *Asc*: F-GCTTGGAGCTCACAAATGACTG; R-CAGCACACTGCCATGCAAAG *Nlrp3*: F-AGGGTCAAAACGCTTTCCCT; R-GAGGTCCAGTTCAGTGAGGC *Nlrc4*: F-GATCTAGGAACGTGGGACGC; R-ATGGATGATCTTTCGGGCGG

*Il18*: F-GAAAGCCGCCTCAAACCTTC; R- GGTTGTACAGTGAAGTCGGC

*Il1b*: F-CTCCAGCCAAGCTTCCTTGT; R-TGATGTGCTGCTGCGAGATT
