# Supplementary figures and images for: Caspase-11-dependent pyroptosis of lung epithelial cells protects from melioidosis while caspase-1 mediates macrophage pyroptosis and production of IL-18
Source: PLoS Pathog. 2018 May 23;14(5):e1007105. doi: 10.1371/journal.ppat.1007105 (PMC5988316; doi:10.1371/journal.ppat.1007105)

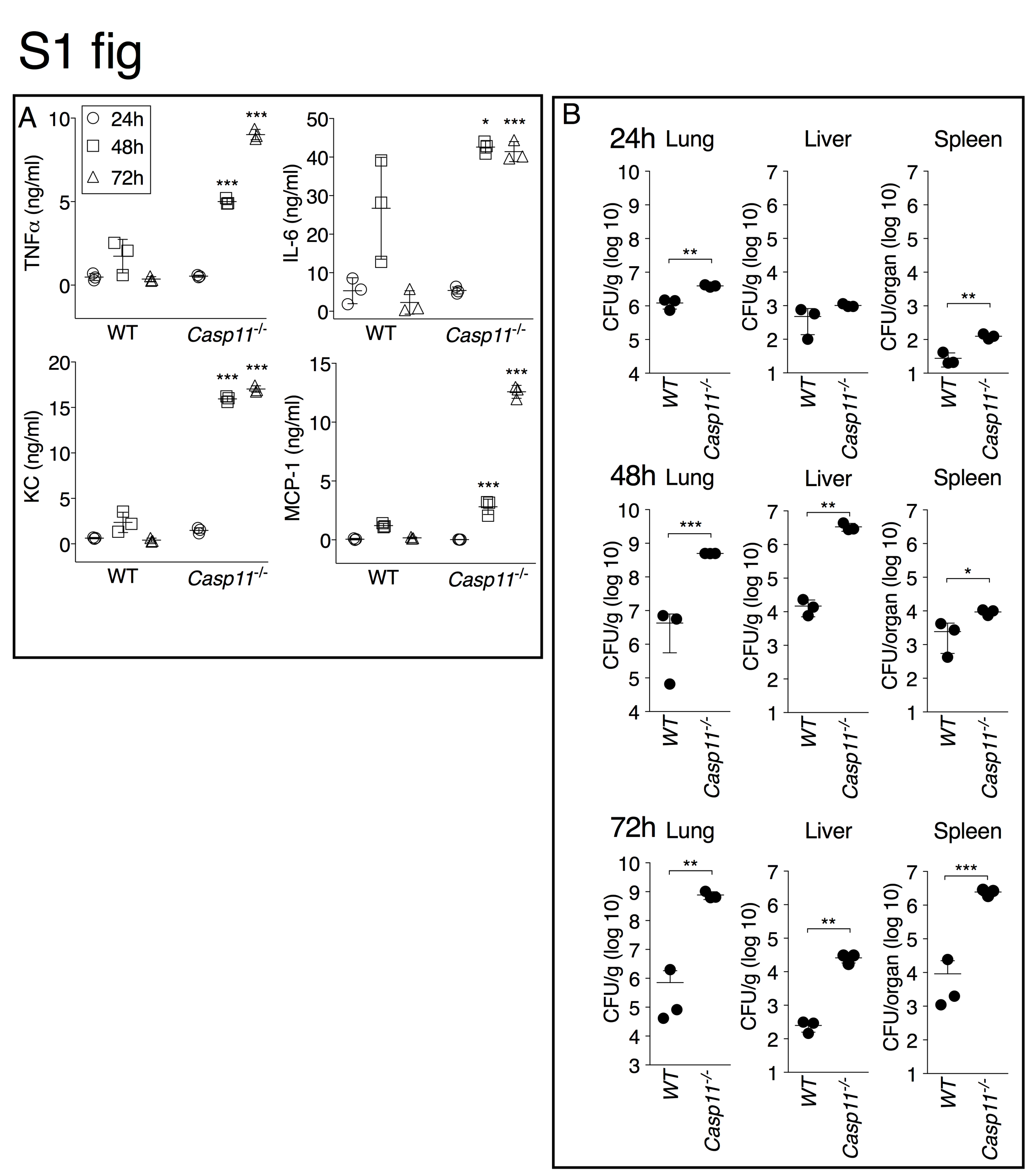

Supplement: S1 Fig — Wild type or Casp-11-/- mice infected intranasaly with B. thailandensis (105 CFU) were sacrificed at the shown time points and cytokine and chemokines levels in BALF (A) or organ bacterial burdens (B) were measured. Data are expressed as mean ± S.D. *p<0.05, **p<0.01. (A) One-way ANOVA, (B) Unpaired t-test. (TIFF) [file ppat.1007105.s002.tiff]

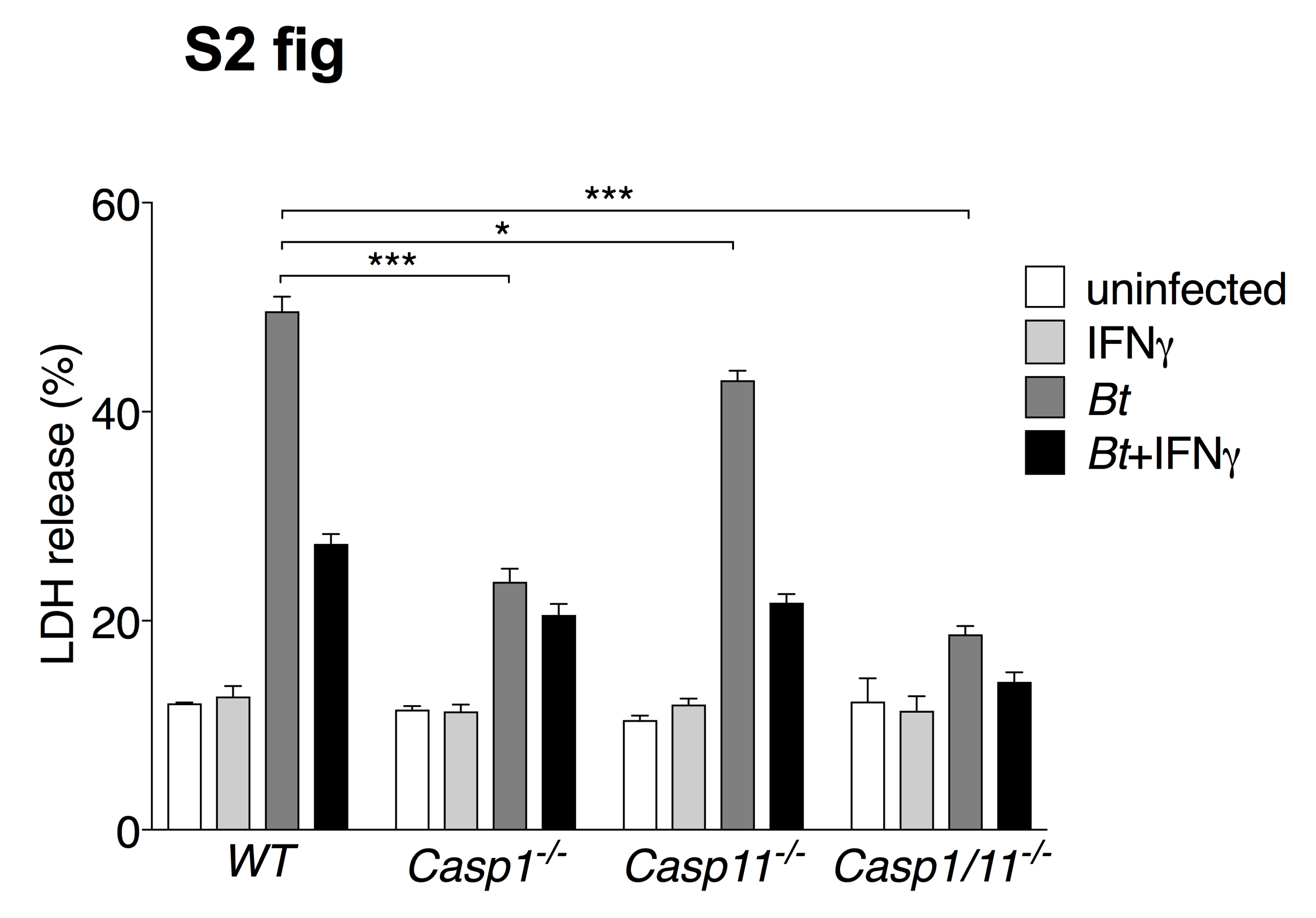

Supplement: S2 Fig — BMDC were treated with IFNγ (100 ng/ml) and simultaneously infected with B. thailandensis (MOI 50). LDH release was measured 6 hours p.i. *p<0.05, **p<0.01. One-way ANOVA. (TIFF) [file ppat.1007105.s003.tiff]

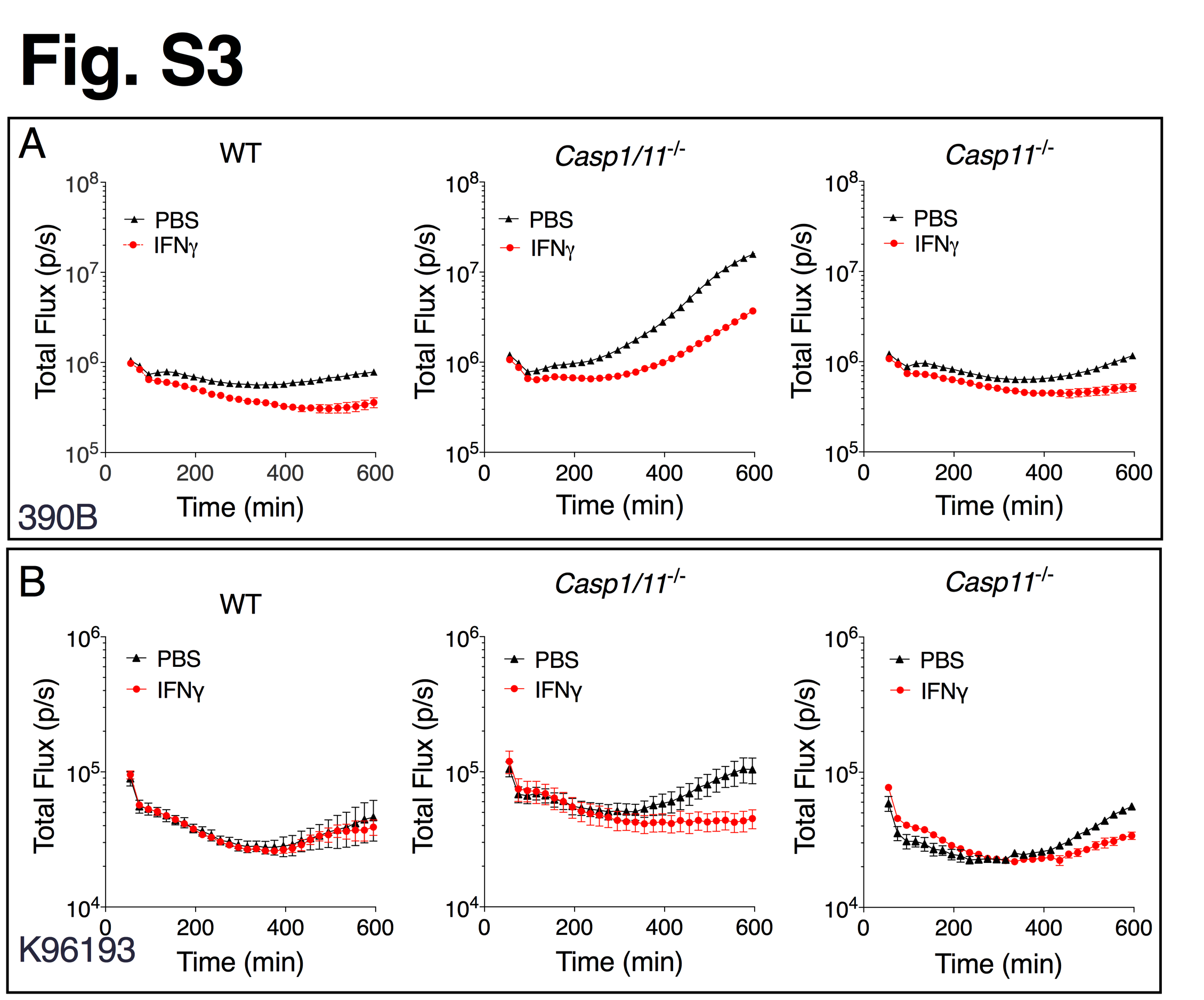

Supplement: S3 Fig — BMM of shown genotype were treated with IFNγ (100 ng/ml) and infected with light emitting B.pseudomallei clinical isolates 390b (A) or K96243 (B) (MOI 10). Bacteria replication (as measured by light emission) was monitored for 600 minutes post infection. One representative experiment of two is shown. (TIFF) [file ppat.1007105.s004.tiff]

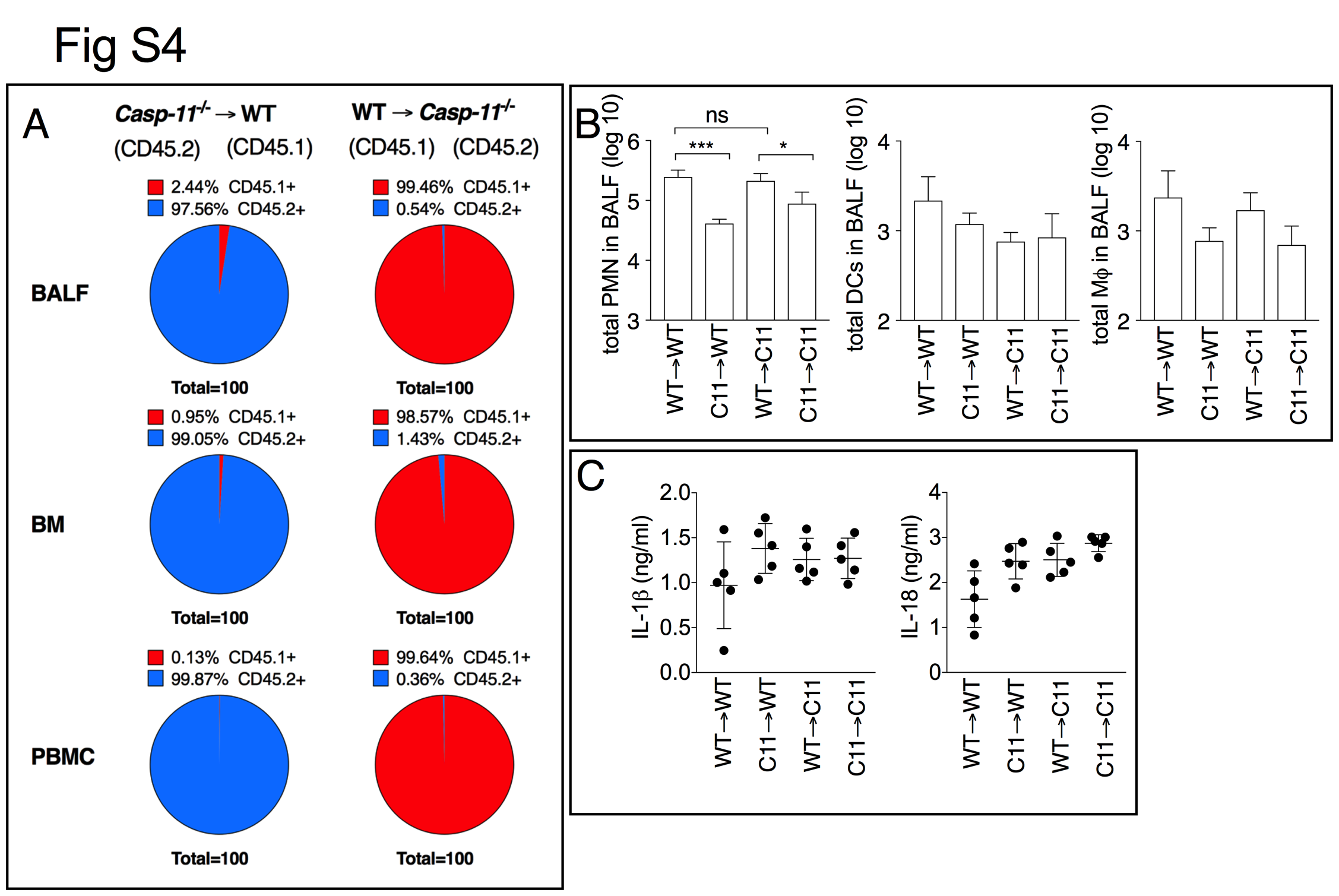

Supplement: S4 Fig — (A) Efficiency of bone marrow reconstitution was measured in BALF, bone marrow (BM), and PBMC by staining CD45.1- and CD45.2-positive cells. (B) Total number of neutrophils, DCs, and macrophages in BALF of infected mice from Fig 3. (C) IL-1β and IL-18 were measured in BALF of infected mice from Fig 3. (TIFF) [file ppat.1007105.s005.tiff]

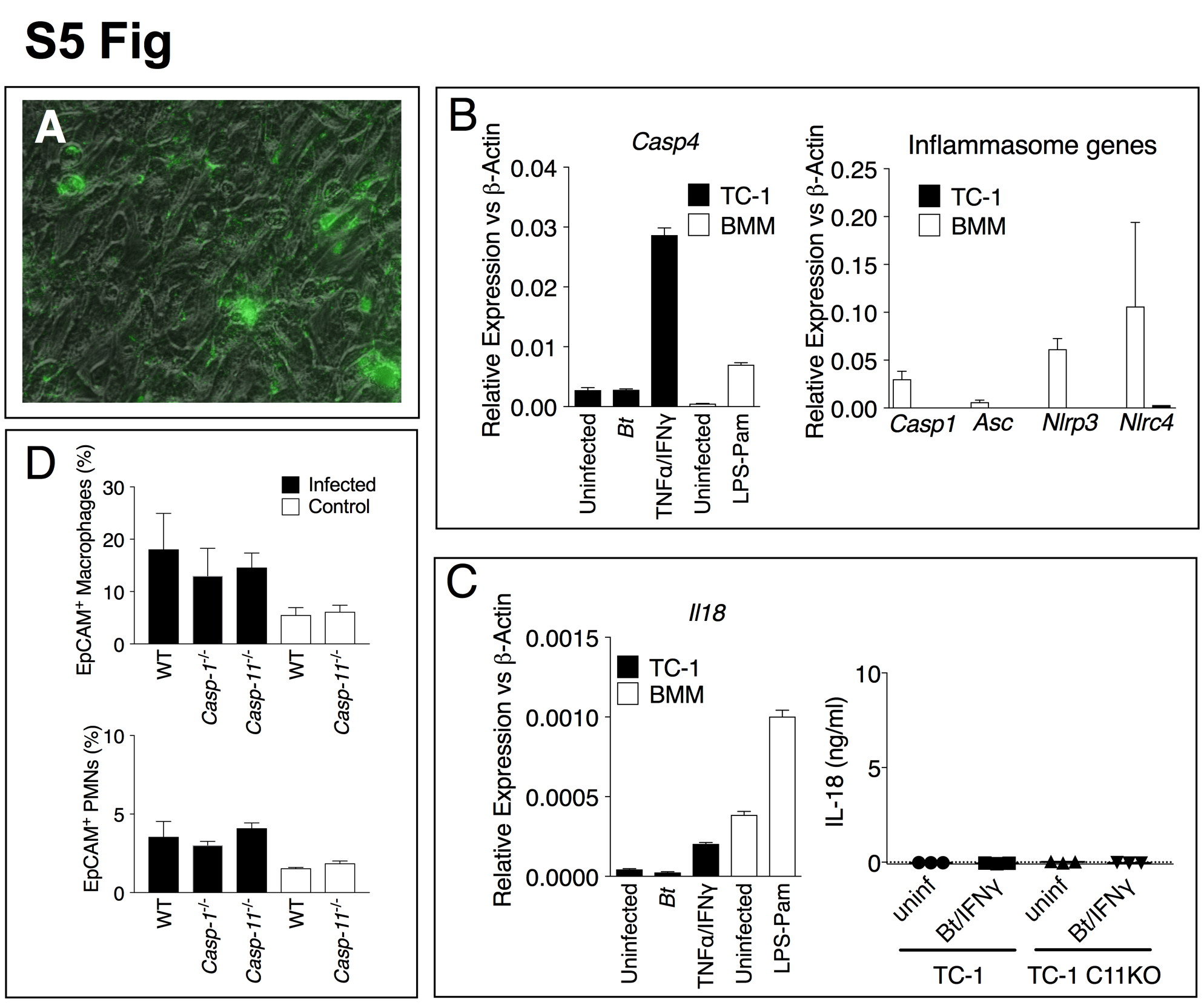

Supplement: S5 Fig — (A) TC-1 cells were infected with GFP-expressing B. thailandensis (MOI 50). (B) Relative expression of Casp4 and canonical inflammasome components in TC-1 cells stimulated with TNFα (50 ng/ml) and IFNγ (100 ng/ml) for 8 hours or in BMM. (C) Expression of Il18 mRNA or measurement of IL-18 in TC-1 conditioned supernatants. (D) Macrophages and neutrophils obtained from control or infected mice were stained for EpCAM and analyzed by flow cytometry. (TIFF) [file ppat.1007105.s006.tiff]

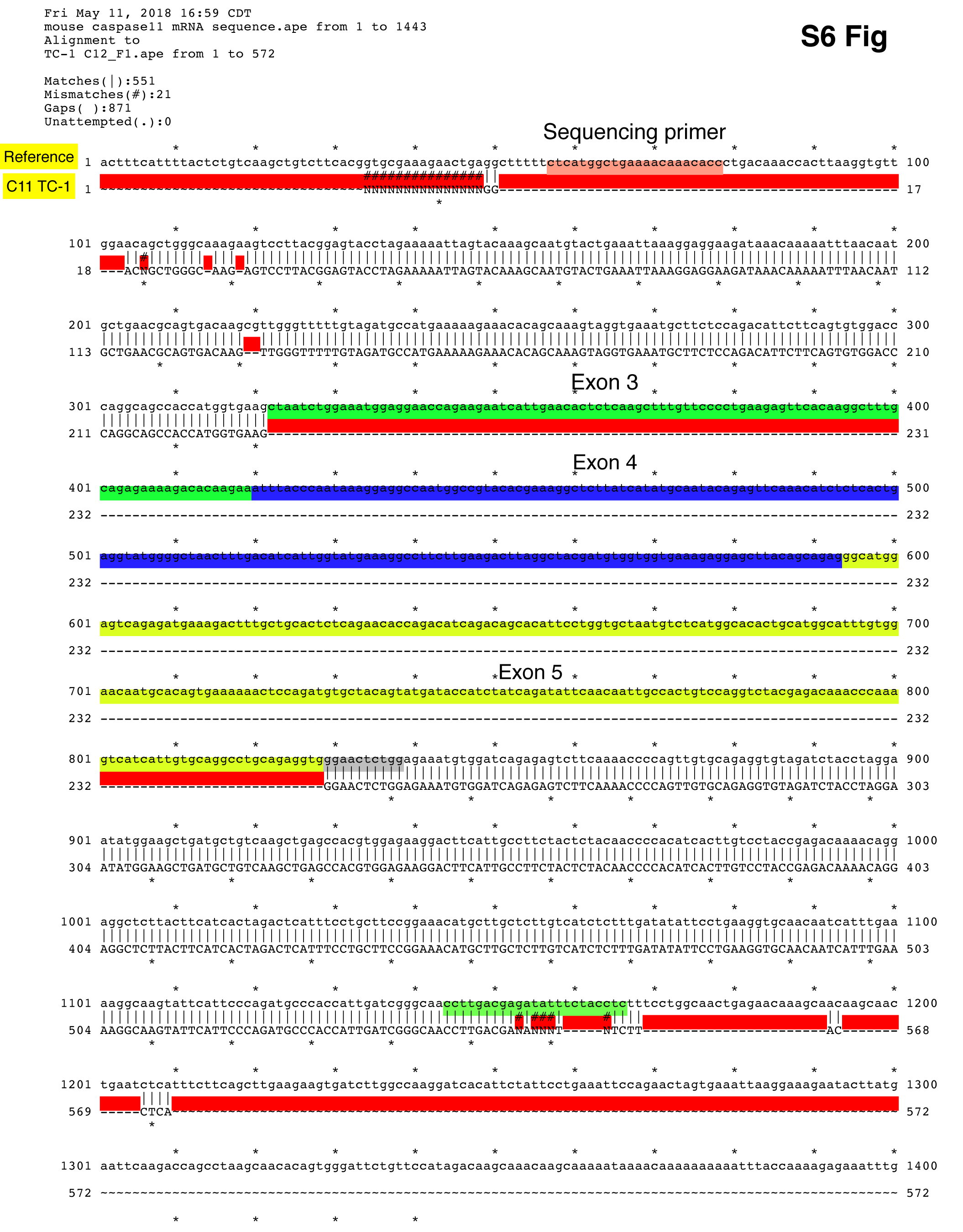

Supplement: S6 Fig — Sequence alignment of reference and targeted Casp4 cDNA showing deletion of exons 3, 4, 5 in TC-1 C11 KO. (TIF) [file ppat.1007105.s007.tif]
